# Notes and News

**Published:** 1894-10-27

**Authors:** 


### Notes and News.

COLLECTED AND COMMUNICATED.

Speaking at the Harveian Society last Thursday, Mr. Jacobson drew attention to the possible influence of mitral stenosis as predisposing to hæmorrhage after operation. The question of anæsthetics in such cases was also discussed, and it was suggested that the tendency of ether to produce pulmonary embarrassment might, in cases where the lung was already engorged in consequence of a narrow mitral orifice, produce such fulness of the systemic veins as to lead to hæmorrhage. At any rate, the point is worth bearing in mind, especially in view of the fact that some of those who have a certain amount of mitral narrowing show so few direct symptoms of the condition that the question of heart disease hardly arises. The relation between this lesion and recurrent hæmorrhage deserves working out by house surgeons and surgical registrars.

A correspondent kindly calls our attention to an omission from the article on Hospital Finance, which appeared in these columns on page 31, on October 13th. The writer failed to point out that in the comparison made between the cost per bed at St. Mary's and University Hospital, no allowance was made for the fact that at St. Mary's in-patients are provided with everything, whereas at University Hospital they have to provide their own tea, butter, sugar, a change of linen, besides having to pay for their own washing. At University Hospital, too, each patient has to bring a teacup and saucer, spoon, knife and fork, soap and towel, as well as the food mentioned. We understand that an energetic house physician once succeeded in doing away with this objectionable system, as far as the children's ward was concerned, as it leads to patients keeping their own butter, jam, tea, and sugar amongst a miscellaneous collection of other properties, in the lockers by the beds. We hope that not only at University Hospital, but at every other hospital where this old bad system still prevails, some influential member of the staff or some Governor will at once cause an agitation in favour of the abolition of a system which ought not to be permitted for one moment to continue in connection with a hospital which has any pretensions to be regarded as of the first rank.

A large gathering assembled at the Hospital for Consumption at Ventnor recently to witness the unveiling of a memorial portrait of the late founder of the institution, Dr. Arthur Hill Hassall.

Those who believe in "Nature's" cures, to the abhorrence of drugs, will welcome the assertion of an American doctor, who affirms that the best remedy for a cough is a spoonful of moderately hot water. At any rate the experiment is simple and within the reach of all.

A new hospital, the Lady Griselle Baillie Memorial Hospital, was opened in Edinburgh last week. The hospital is to accommodate twenty patients, and the total cost of erection is estimated at £3,500. It is to be worked in connection with the Church of Scotland Deaconesses' Institution, and will be especially adapted to the training of nurses working for this community.

Much satisfaction is evinced throughout Germany at the selection of a Berlin physician as medical attendant to the unfortunate Czar. Professor Leyden has left his patients in charge of a colleague, and has been granted leave of absence from the University in order to join his illustrious patient at Livadia, though it is to be feared on his arrival he met with no hearty welcome on the part of his Russian colleagues.

At the last meeting of the delegates of the Metropolitan Hospital Saturday Fund, a report of the council was read, and showed that the collection for 1894 amounted to £11,117 9s. 7d., of which £6,275 11s. 10½d. was realized by the workshops' collection, and £4,526 6s. 10d. by the streets' collection. The diminution of the latter as compared with 1893 was £450, and was accounted for by bad weather during the collection.

The question of admitting lady medical students into the Dundee Royal Infirmary came before the directors of the institution at the last meeting. Lady students at present attend the course of instruction in common with the male students at University College; they now ask to receive practical instruction at the infirmary also. A section of the directors attending the meeting expressed strong disapproval of mixed instruction, and considering the small number present and the absence of Dr. Raw, the medical superintendent, it was agreed to defer a definite decision, and that enquiries should be made as to the practice which obtained in other infirmaries, and to secure general information on the matter.

The new buildings at the Royal Free Hospital were formally inspected by the Lord Mayor and the Lady Mayoress on Wednesday, 24th inst. Members of the hospital committee and others interested were present in the old Board room to receive the guests. Being himself a member of the committee, the Lord Mayor was able so speak from personal experience of the work done by what he rightly named "a most noble charity." Mr. W. T. Pritchard was in the chair, and the proceedings at this informal opening were very short, and were followed by an inspection of the wards, laundry, dispensary. and the new casualty rooms. The accommodation for the resident staff is greatly improved, and the small rooms for isolation are particularly well adapted for the purpose. Arrangements are in progress for an official opening ceremony when the whole of the building is completed.

